# Emergence of the G118R Pan-Integrase Resistance Mutation as a Result of Low Compliance to a Dolutegravir-Based cART

**DOI:** 10.3390/idr14040053

**Published:** 2022-06-22

**Authors:** Helene Mens, Lasse Fjordside, Jannik Fonager, Jan Gerstoft

**Affiliations:** 1 Rigshospitalet, Department of Infectious Diseases, 2100 Copenhagen, Denmark; lasse.fjordside@regionh.dk (L.F.); jan.gerstoft@regionh.dk (J.G.); 2 Viruses and Microbial Special Diagnostics, Statens Serum Institut, 2300 Copenhagen, Denmark; fon@ssi.dk

**Keywords:** HIV-1, integrase inhibitors, drug resistance

## Abstract

HIV-1 resistance towards integrase inhibitors is a potential threat of the success of HIV-1 combination treatment. G118R is a rare drug resistance mutation conferring pan-integrase resistance. Here, we describe the occurrence of G118R in a HIV-1 subtype-B-positive individual with major compliance problems, detected while the patient was on dolutegravir-based cART. We speculate the pre-selection of M184I/V aided the occurrence of G118R in this case, and discuss the robustness of dolutegravir-based therapies.

## 1. Introduction

Dolutegravir is a second-generation integrase inhibitor (InSTI), low in side-effects and drug-to-drug interactions, and with a once-daily formulation and a high genetic barrier for drug resistance development. Further, dolutegravir in two-drug regimens has proven to be non-inferior to the traditional three drug regimens in several randomized trials [1,2,3,4], and American, as well as European, guidelines now include DTG 2DRs for both treatment-naïve and virally suppressed patients.

The high genetic barrier to resistance for dolutegravir is partly explained by the requirement of more than one mutation in the integrase gene to reduce drug sensitivity. As an example, the Q148H/R/K alone does not reduce dolutegravir susceptibility notably. However, in combination with G140S and E138K, susceptibility is reduced 10–20-fold. Unlike the Q148H/R/K pathway, the G118R mutation alone confers high-level resistance, and is associated with a significant reduction of HIV replication capacity [5,6,7]. G118R is rarely observed; in a study of 1100 patients failing a INSTI-based regimen in France, it was not reported [8]. It has only been reported in a handful of cases failing dolutegravir [9,10,11,12,13,14,15], mostly non-B subtypes. At least in two of the cases, the selection was facilitated by the GGA natural polymorphism at codon 118 (1.5% prevalence) requiring only one mutation to change from GGA (G) to AGA (R). However, most clinical isolates harbor the GGC polymorphism at codon 118 (79% of all strains, and 91.8% of all subtype B), which requires two mutations, GGC to AGA, to change from G to R.

## 2. The Case Report

Here, we report the selection of pan-integrase resistance mutation G118R in a treatment-experienced 50-year-old male with a history of major compliance problems. The patient was infected with HIV-1 subtype B through blood products in the 1980s. He had been exposed to nucleotide-based therapies in the 1990s, and had previously failed NNRTI, protease, and first generation INSTI-based regimens, rendering the M184V mutation in the reverse-transcriptase gene. The patient failed Triumeq (abacavir, lamivudine, and dolutegravir) following years of non-compliance and documented viral loads of around 200 copies/mL on several occasions. In August 2020, the occurrence of the G118R mutation was documented, conferring pan-integrase resistance. Hereafter, cART was changed to Symtuza (cobicistat, darunavir emtricitabine, and TAF). The patient gained virus control. However, with the accumulated drug resistance, future therapeutic options are currently very limited. All genotypic drug resistance test were performed on viral RNA extracted from plasma. Sequences were obtained by standard RT-PCR targeting the integrase coding region of HIV-1, followed by Sanger sequencing [16], or targeting the region containing the protease and reverse transcriptase encoding genes using either the Viroseq kit as recommended by the manufacturer, or by using an in-house RT-PCR assay. GenBank accessions numbers (ON653600-03).

**Figure 1:**
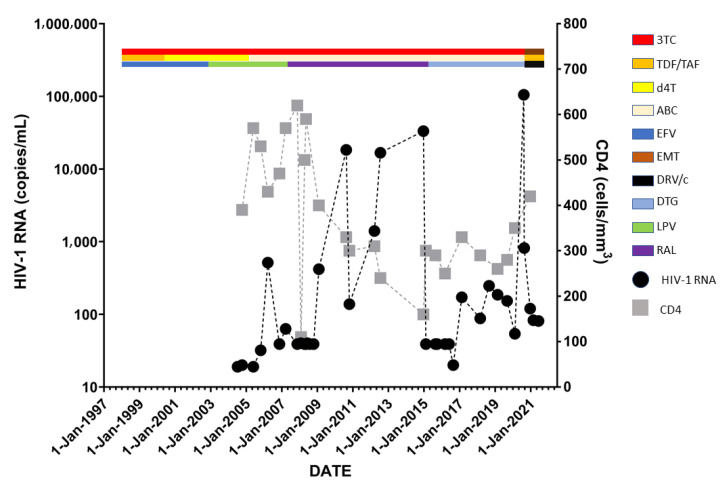
Treatment history and HIV status with HIV-1 RNA in copies/mL and CD4 cell counts in cells/mm^3^.

**Figure 2:**
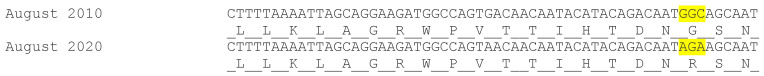
The evolution at site 118 of the integrase gene (highlighted in yellow) at nucleoside and amino acid level GGC (G) to AGA (R).

Treatment history and HIV status are shown in Figure 1. Figure 2 illustrates the evolution at site 118 of the integrase gene. Table 1 shows the three genotypic drug resistance tests performed during the course of antiretroviral treatment for this patient.

## 3. Discussion

Our case highlights the risk of developing pan-integrase resistance in patients with non-complacence and non-optimal NRTI backbones. The M184V/I is one of the most common NRTI-associated mutations in HIV-1 infected patients, and is typically selected in patients failing lamivudine (3TC) or emtricitabine (FTC). The presence of M184V/I reduces the susceptibility to these drugs by more than 100-fold, and additionally causes impaired efficacy to abacavir (ABC). Although a large study from five European cohorts did not find excess viral failure upon switching to Triumeq (abacavir/lamivudin/dolutegravir) in patients with documented M184V/I mutation [17], such studies may be biased by a relative low observation time (288 days), and biased towards switching highly compliant patients to a single tablet regimen. We speculate that the pre-selection of M184V may have facilitated the development of G118R in this case. A very similar case has been reported [18]. In line with these considerations, the TANGO trial [19] found the combination of M184V/I and at least one TAM and previous failure on an INSTI-based regimen to predict failure upon switching to lamivudine and dolutegravir, highlighting the importance of an optimal NRTI backbone for virologic success. Although the overall finding from the NADIA trial [15] was noninferiority between dolutegravir and ritonavir boosted darunavir (darunavir/r) in combination with either tenofovir or zidovudine as second-line treatment, four patients failing dolutegravir had confirmed major drug resistance mutations versus none failing darunavir/r. Two failed with the G118R mutation, suggesting darunavir/r may be safer long-term in the setting of NRTI resistance and adherence problems. However, data beyond 48-weeks are needed to confirm this. Thus, drug history and previous drug resistance tests should be carefully revised before changing antiretroviral treatment to a dolutegravir-based regimen.

## Figures and Tables

**Table 1:** Results of genotypic drug resistance tests over the course of treatment.

Date	Major PI	Other PI	Major RT	Other RT	Major IN	Other IN
April 2003	none	M36I, L63P, I93L, I151IV, N37D, I62V, I72V	K103N, V179DV, M184V, P225H	K20R, V35M, T39S, R83K, E169D, T200A, Q207E, K238T, A288S, I293V, S322T, Q334L, F346Y	NA	NA
August 2010	none	I15IV, M36I, N37D, I62V, L63P, I72EV, I93L	K103KN, V106VIM, V179VD	K20R, V35VM, T39S, R83K, E169D, P170PL, T200A, Q207E, F214FL, I257IL, A288S, I293V, S322T, Q334L	T66TP	S39C, D41DE, V75VL, A76AS, H78HY, V79VA, K111T, S255T
August 2020	none	M36I, N37D, K43R, I62V, L63P, I72E, I93L	M184V, V179D	K20R, V35M, T39S, R83K, I135L, E169D, G196E, T200A, Q207E, A288S, I293V, I309L, S322T, Q334L	G118R	S39C, K111T, V201VI, S255T

## Data Availability

HIV-1 sequences have been submitted to GenBank accessions numbers ON653600-03.
